# Functional and molecular insights into muscle proprioceptors

**DOI:** 10.7554/eLife.106803

**Published:** 2025-11-28

**Authors:** Francois Lallemend, Prach Techameena, Saida Hadjab

**Affiliations:** 1 Department of Neuroscience, Karolinska Institutet Stockholm Sweden; 2 Laboratory of Neurobiology of Pain & Therapeutics, Department of Neuroscience, Karolinska Institutet Stockholm Sweden; National Institutes of Health United States; National Institute of Neurological Disorders and Stroke United States

**Keywords:** somatosensory, peripheral nervous system, mouse, proprioception, molecular biology, cellular biology

## Abstract

Proprioception, the innate ability to perceive body positions and movements, enables us to perform daily activities without thinking about it. In mammals, this process primarily involves the activation of three types of proprioceptive neuron (PN) endings in muscles (Ia and II-PNs) or tendons (Ib-PNs). However, recent research indicates that these cardinal classes exhibit molecular diversity that likely reflects differences in connectivity, morphology, and activity patterns, contributing to the detection of various kinematic parameters. In this review, we summarize the properties and functions of PNs and propose a comprehensive cell-type classification. By systematically mapping functionally relevant molecular markers to specific PN subtypes, we establish a tentative, yet insightful taxonomy based on their functional characteristics. This foundational work lays the groundwork for future research aimed at elucidating the distinct physiological properties of each PN subtype and their interactions within central motor circuits. Understanding these nuances will be critical for advancing our knowledge of sensorimotor circuitry and its role in movement control.

## Introduction

**Figure 1.**
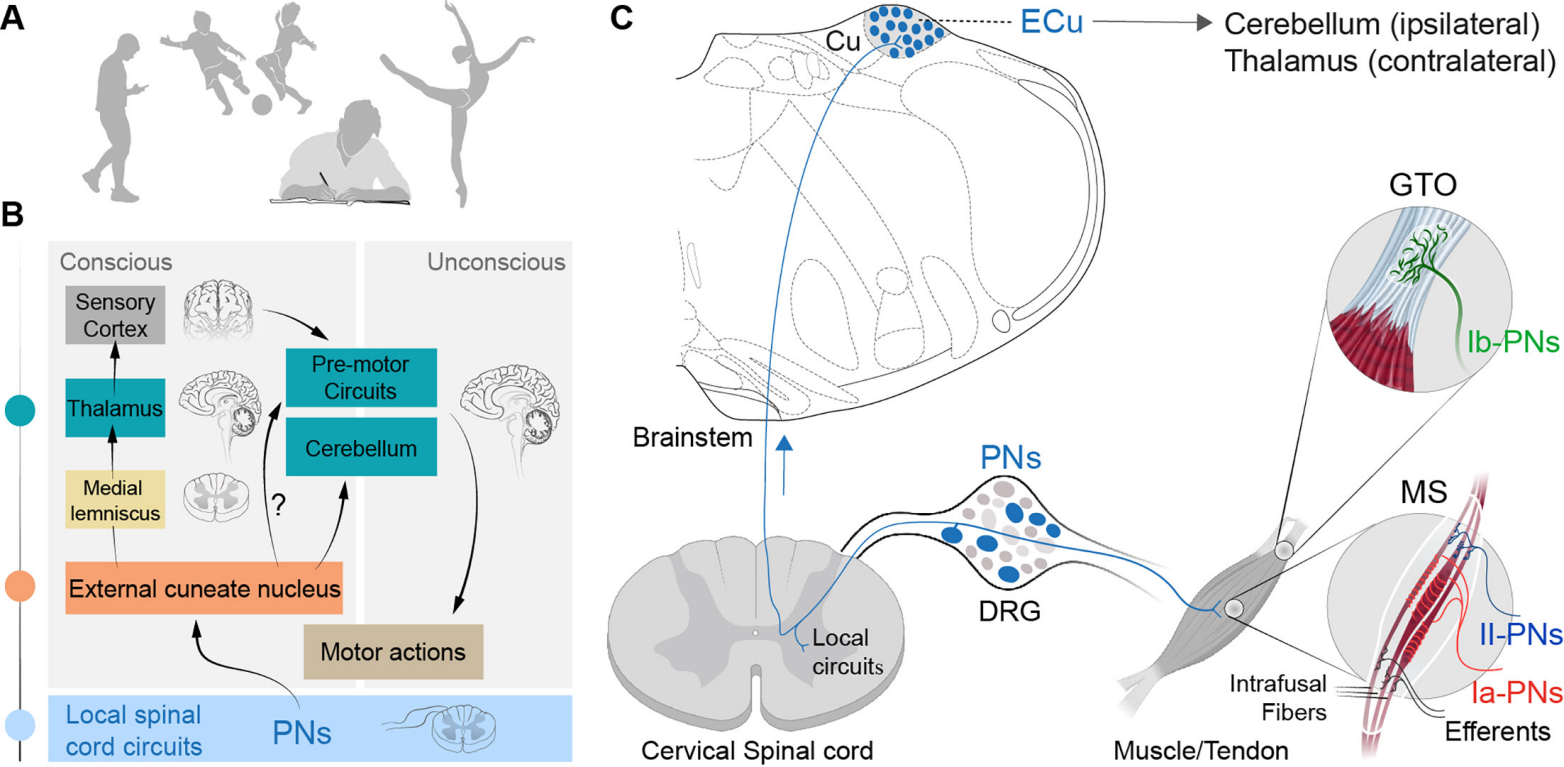
Overview of the dorsal root ganglia (DRG) muscle proprioceptive system. (**A**) Examples of behavior that rely on muscle proprioceptive feedback for proper execution. (**B**) Partial schematic representation of the ascending proprio-brainstem pathways through the external cuneate nucleus (ECu) and supporting skilled forelimb behavior; the question marks indicate putative pathway. (**C**) Incomplete circuit diagram of the proprioceptive-CNS network for movement control, showing local connections with interneurons (not shown), and emphasizing the ECu as a key processing hub for supraspinal proprioceptive feedback. The schematic also illustrates the peripheral terminations of proprioceptive neurons (PNs) within skeletal muscles and highlights the classic, distinct subtypes of PNs. Abbreviations: Cu, cuneate nucleus; GTO, Golgi tendon organ; MS, muscle spindle.

Coined by Sherrington, 1910, ‘proprioception’ refers to a range of receptors that provide information concerning limb positions, motions, tension, effort, and balance (Proske and Gandevia, 2012; Figure 1). Although essential, proprioceptive sensations often go unnoticed, seamlessly integrated with motor actions. However, this ‘sixth sense’ plays a crucial role in daily function, as evidenced by its impact on spinal cord motor networks and the impaired movement observed in humans and animals that have lost proprioception (Akay et al., 2014; Chesler et al., 2016; Conway et al., 1987; Hilde et al., 2016; Kiehn, 2016; Koch et al., 2017; Lajoie et al., 1996; Martino et al., 2014; Santuz and Akay, 2023; Santuz et al., 2022; Takeoka and Arber, 2019; Takeoka et al., 2014; Woo et al., 2015).

In mammals, proprioceptive sensations originate from peripheral sensory organs located in muscles (muscle spindles) and tendons (Golgi tendon organ), detecting muscle contractions and load, respectively (Proske and Gandevia, 2012; Figure 1). They are conveyed through proprioceptive neurons (PNs) of the dorsal root ganglia (DRG) which send signals to the spinal cord and the brainstem through highly myelinated axons (Kalambogias and Yoshida, 2021; Lallemend and Ernfors, 2012; Loutit et al., 2021; Shadrach et al., 2021; Versteeg et al., 2021). This muscle sensory feedback provides instant updates on the actual body and limb positions that are pivotal for setting the initial motor commands for smooth and coordinated movements, body posture, and motor learning (Matthews, 1964; Proske and Gandevia, 2012). Central commands for any motor task, such as reaching or locomotion, must also continuously incorporate changes in the position of the body parts. Thus, proprioceptive pathways conveying PNs-detected muscle and tendon information from periphery to the central nervous system include secondary neurons in the spinal cord for local modulation of motor output and ascending projection pathways (Gradwell et al., 2024; Hilde et al., 2016; Koch et al., 2017; Marasco and de Nooij, 2023). Ascending projections relay proprioceptive information to higher brain centers, including the cerebellum and the external cuneate nucleus (ECu) in the medulla (Loutit et al., 2021; Marasco and de Nooij, 2023; Figure 1). Proprioceptive sensory streams are also transmitted directly to the ECu along the cuneate fasciculus, which also projects to the cerebellum (Marasco and de Nooij, 2023; Versteeg et al., 2021). While proprioceptive inputs to the cerebellum contribute to subconscious corrections of limb and body posture, proprioceptive data within the ECu also ascends to the somatosensory cortex through the dorsal column medial lemniscus pathway, enabling conscious perception (Alonso et al., 2023; Marasco and de Nooij, 2023).

Proprioceptive information relies on an intricate network of diverse origins, encompassing a variety of muscle types, an assortment of sensory organs and receptors, along with a multifaceted central processing framework. This framework underpins every aspect of proprioception, spanning from simple spinal reflexes to complex supraspinal reflexes, and the planning and execution of voluntary movements and conscious perception. However, despite its complexity, proprioception primarily operates in the background, enabling individuals to stay attuned to other senses while in motion, which might explain the lower attention it has received in comparison to other somatosensory sensations like pain and touch (Abraira and Ginty, 2013; Handler and Ginty, 2021; Kupari and Ernfors, 2023).

In this review, we summarize recent efforts in defining the biological basis governing the identity and organization of PNs of the DRG. Interested readers should consult recent reviews and critical works concerning molecular plasticity and development of PNs, as well as their ascending pathways (Balaskas et al., 2019; Catela et al., 2015; de Nooij and Zampieri, 2023 Dietrich et al., 2022; Lallemend and Ernfors, 2012; Loutit et al., 2021; Marasco and de Nooij, 2023; Versteeg et al., 2021; Wang et al., 2019a; Wang et al., 2019b; Wu et al., 2021). Also, PNs of the mesencephalic trigeminal nucleus, with cell bodies in the brainstem and projections to peripheral targets like jaw muscle spindles, will not be discussed further (Lazarov, 2002), as this review focuses on DRGs.

## Canonical characteristics of proprioceptors

Muscles, innervated by fast-conducting Aα fibers, house receptors crucial for proprioception. Among these, muscle spindles (MSs) consist of thin intrafusal muscle fibers enclosed within a capsule (Bornstein et al., 2023; Proske and Gandevia, 2012). Mechanosensitive afferent fibers wrap around the intrafusal muscle fibers and detect stretching, with their firing rate correlating to changes in muscle length (Proske and Gandevia, 2012). The sensitivity of MSs can be finely adjusted by the contraction of these intrafusal fibers, which are innervated by γ-motor neurons (fusimotor efferents) (Boyd, 1976; Boyd et al., 1977; Crowe and Matthews, 1964; Dimitriou, 2022; Olson et al., 2024). In contrast, extrafusal muscle fibers, responsible for generating force, are innervated by α-motor neurons (Proske and Gandevia, 2012). A less well-characterized population, the β-motor neurons, innervates both intrafusal and extrafusal muscle fibers and is thought to play a role in coordinating muscle contraction with spindle sensitivity (Boyd et al., 1977; Emonet-Dénand et al., 1970; Jami et al., 1985; Niyo et al., 2024). This population may also have been identified at the molecular level in recent single-cell sequencing studies in mice (Alkaslasi et al., 2021; Blum et al., 2021). Golgi tendon organs (GTOs), positioned between muscles and tendons, sense muscle force rather than length and wield a substantial role in reflex circuits (Akay et al., 2014; Pearson, 2004; Proske and Gandevia, 2012). Additional joint-capsule receptors within joints sense tension and are not discussed here (Macefield, 2021; Proske, 2023). Regarding this, it is worth mentioning that individuals with artificial joints, lacking joint-capsule receptors, can still accurately sense the angle and motion of their prosthetic joints, which would primarily rely on input from MSs (Proske and Gandevia, 2012).

Among PNs, detailed anatomical and electrophysiological studies on various model organisms have previously identified three major functional classes: stretch-sensitive Ia- and II-PNs, which innervate MSs, and force-sensitive Ib-PNs, which innervate GTOs (Hultborn, 2006; Proske and Gandevia, 2012). The central region of MSs intrafusal fibers is innervated by Ia-PNs, while II-PNs and γ-motor neurons innervate their polar ends (Proske and Gandevia, 2012). Ia- and II-PNs relay the rate (velocity) and magnitude of changes in muscle length, respectively (Matthews, 1964; Proske and Gandevia, 2012; Windhorst, 2007). These are commonly named primary (I) and secondary (II) muscle afferents, based on their morphological characteristics (see below) (Lloyd and Chang, 1948; Proske and Gandevia, 2012). Ib-PNs, on the other hand, signal the force the muscle exerts and are also a type of primary (I) afferents. These low-threshold mechanoreceptor (LTMR) muscle afferents display firing patterns that are both common and distinct in response to mechanical stimuli (Hultborn, 2006; Matthews, 1964; Proske and Gandevia, 2012). They all increase their firing rates, which then slowly adapt as the muscle they are associated with is stretched further from one fixed point to another. Additionally, these muscle afferents also fine-tune their firing activity in varying ways based on particular features of the muscle’s mechanical responses. This tuning process enables the conventional categorization of LTMR muscle afferents into the three distinct subtypes that mainly differ physiologically in parameters such as initial burst, deceleration response, silencing during muscle shortening, and stretch sensitization (Hultborn, 2006; Matthews, 1964; Matthews, 1972). The key variables considered are dynamic response, static response, and threshold/sensitivity (Matthews, 1972). Among these, Ia stands out from the others due to its dynamic (velocity) and sensitivity characteristics, while the static (length) response remains similar across subtypes. Indeed, Ia-PNs are more responsive to dynamic stretch than II afferents, and Ib afferents do not respond to stretch (Hultborn, 2006; Matthews, 1964; Matthews, 1972; Proske and Gandevia, 2012). Also, their firing is perfectly entrained to high-frequency muscle vibration; it responds with a high-frequency initial burst at the onset of ramp stretch, falls to lower rates in transition from dynamic to static stretch, and persists but with distinct accommodation during the hold phase of stretch, making the Ia-PNs highly sensitive to velocity (Housley et al., 2024a; Housley et al., 2024b; Proske and Gandevia, 2012). Interestingly, the signaling of dynamic muscle stretch by Ia afferents is significantly more pronounced in rats than in cats, irrespective of the physical variations between the two species (Banks, 2006; Matthews, 1964). Additional research is needed to assess whether the differences in dynamic firing patterns of group Ia afferents represent species-specific adaptations to variations in kinematic and kinetic parameters (Carrasco et al., 2017; Vincent et al., 2017). Conversely, the II-PNs exhibit sensitivity to muscle length and reflect static muscle kinematics. Ib afferents are primarily activated by muscle contraction and show tonic firing during isometric contraction, reflecting steady muscle force (Houk and Henneman, 1967; Jami, 1992; Jansen and Rudjord, 1964).

Although PNs have been broadly classified, the firing responses and properties within groups exhibit considerable variability (Matthews, 1964), with more overlap observed between Ib and II afferents. Furthermore, delineating the two kinds of spindle afferents according to their response characteristics is complicated by the influence of fusimotor control on both, affecting spindle sensitivity and afferent behavior. We will not delve into this matter and instead refer to existing literature that explores the role of the γ-motor neurons in modulating the tuning of MSs (Banks, 2024; Boyd, 1976; Boyd et al., 1977; Crowe and Matthews, 1964; Dimitriou, 2022).

With only a few muscles as exceptions, MSs are present in every skeletal muscle examined across various mammals. However, their density significantly differs among muscle types (Banks, 2006). MSs are especially prominent in muscles involved in fine movement regulation, including those that control the hands and digits, or the neck (Banks, 2006). Another variation lies in the diverse complexity of MSs. This complexity refers to the number of type II afferent endings per MS. On average, MSs contain one secondary ending (Matthews, 1972). However, specific muscles can exhibit significant differences in the relative numbers of secondary to primary endings within each MS. It has been proposed that complex MSs (with multiple secondary endings) are particularly abundant in tonic muscles (e.g. biceps brachii), while simple MSs (with no secondary ending) are more commonly found in phasic muscles (e.g. triceps brachii) (Matthews, 1972; Wu et al., 2021). These characteristics are likely to vary between species, potentially influencing muscle type-specific sensory feedback and tuning the overall proprioceptive control of movement.

Although GTOs have been identified in the muscles of the limb and neck, they are probably not present in the majority of axial muscles in the thoracic segments, except for the diaphragm (Jami, 1992; Wu et al., 2021); there are conflicting results, however, regarding the presence of GTOs in the intercostal muscles (Holt et al., 2002; Wu et al., 2021). Moreover, GTO counts within individual muscles are seldom detailed, and systematic assessments are lacking. Their quantity is typically fewer than that of MSs, exhibiting significant variability. An interesting observation is the apparent relatively low ratios of GTOs to MSs in intrinsic hand muscles (Matthews, 1972), indicating a decreased necessity for GTO feedback from muscles engaged in precise motor control.

Another key factor that distinguishes the three types of muscle LTMR fibers, serving as an anatomical classification, is their size (Lloyd and Chang, 1948; Matthews, 1972). This variation in fiber diameter is closely tied to two physiologically significant parameters: conduction velocity and electrical excitability (Erlanger and Gasser, 1930; Hursh, 1939). Sherrington initially described a wide range of muscle afferent diameters, spanning from 1.5 to 20 µm (Sherrington, 1894), a distribution later confirmed by subsequent studies (Lloyd and Chang, 1948; Matthews, 1972). This distribution is trimodal, reflecting the categorization of fibers into groups Ia, Ib, and II, arranged from largest to the smallest diameter. While some overlap and variability are observed – especially between different muscles and species – the trimodal distribution remains a consistent feature of muscle afferent classification (Matthews, 1972). The only clear conclusion drawn from these data is that most of the largest fibers, which exhibit greater dynamic sensitivity, likely originate from Ia endings, while the smallest fibers are typically associated with II endings (Matthews, 1972). However, there are no clear-cut boundaries that separate these groups based on electrophysiological or anatomical parameters. As a result, a substantial representation of fibers with intermediate characteristics exists, raising the possibility that further subdivisions of PNs into functionally distinct subgroups may be necessary.

## Comprehensive classification of PN subtypes

In recent years, remarkable progress has been made in the collection and computational examination of transcriptomes from hundreds to thousands of individual cells, thanks to the advancements in single-cell RNA sequencing technology. Given that the characteristics and traits of a particular cell stem from its distinct gene expression pattern, this technology has successfully delivered an extensive molecular analysis of the diversity of neuron cell types, not only in the central nervous system but also in the peripheral nervous system (Bhuiyan et al., 2024; Kupari et al., 2019; Morarach et al., 2021; Petitpré et al., 2018; Siletti et al., 2023; Yao et al., 2023; Yu et al., 2024). This method has been effectively applied for the examination of DRG neurons heterogeneity in various model organisms (Bhuiyan et al., 2024; Sharma et al., 2020; Yu et al., 2024). It has contributed to the development of comprehensive molecular and cellular atlases, enhancing our understanding of somatosensory neurons and their differentiation (Faure et al., 2020; Lu et al., 2024; Sharma et al., 2020). However, most of the diversity was identified within the nociceptive and skin-LTMR lineages, with minimal to no diversity observed within the PN population, certainly because of the inherent challenge of molecularly profiling low-abundance cell populations. To meet this challenge, recent studies employed mouse genetic strategies utilizing fluorescent-based reporter proteins to enhance the representation of adult PNs and investigate their diversity (Oliver et al., 2021; Wu et al., 2021). Even though both studies revealed a greater diversity of PNs than previously understood, their conclusions regarding the identification of new subtypes slightly differed. Importantly, there did not appear to be discrepancies in the cell types themselves, but rather in the number of cell types identified, which likely influenced the interpretation of the findings. However, by employing an unbiased assignment of the datasets from both studies using machine learning algorithms, along with cross-comparative analysis of gene expression patterns using the label transfer method and neural network-based probabilistic scoring modules, we were able to pinpoint corresponding cell types between the datasets (Figure 2—figure supplement 1). Given that Wu et al.’s nomenclature exhibited the least noise and highest prediction scores, we adopted this nomenclature as the default and created a comprehensive reference atlas of PN subtypes from eight different sources (Jung et al., 2023; Oliver et al., 2021; Qi et al., 2024; Renthal et al., 2020; Techameena et al., 2024; Wang et al., 2021; Wu et al., 2021; Zeisel et al., 2018; Box 1).

Box 1. **Proprioceptive neurons (PNs): a comprehensive cell-type classification.**

To construct a comprehensive single-cell atlas of mouse muscle PNs, we utilized data from eight single-cell or single-nucleus RNA sequencing datasets obtained from various sources (see Materials and methods for more details and accession numbers). These datasets were preprocessed following the thresholds and criteria outlined in their respective original studies. PNs were identified based on the co-expression of specific marker genes: *Pvalb* (parvalbumin), *Runx3*, *Ntrk3* (TRKC), *Etv1* (ER81), and *Whrn* (whirlin). As an initial step in our analysis, we integrated PNs from the datasets of Wu et al. and Oliver et al. (panel A). This integration focused on reconciling the nomenclature systems used in these datasets which were generated using the Smart-seq platform, known for its higher transcript coverage (Ding et al., 2020). During this process, we observed that the nomenclature from Wu et al. could effectively explain the diversity of cell types described in Oliver et al., establishing it as a reference framework for characterizing the cellular heterogeneity of PNs (panel A). Subsequently, we extended our analysis to classify PNs across the remaining datasets, applying the nomenclature system established by Wu et al. To achieve this, we conducted a series of integrative analyses using the scVI and scANVI frameworks. These approaches allowed us to integrate the datasets in both unsupervised and semi-supervised manners, with and without the inclusion of cells from Wu et al. (panels B, C, and Materials and methods section). Finally, we assessed the quality of dataset integration by examining the expression patterns of canonical markers for each PN subtype (panel A). This evaluation confirmed the robustness of the integration and validated the utility of the Wu et al. nomenclature system in describing the diversity of mouse muscle PNs. We then extracted key transcriptional features that may help explain and predict the cellular properties of PN subtypes. Transcriptomic data must be interpreted with caution and validated experimentally to avoid generating misleading hypotheses. Despite this caveat, its widespread availability and ease of analysis have enabled numerous recent studies to infer cell-to-cell communication and related features directly from gene expression data, providing testable hypotheses across various fields (Armingol et al., 2021). This approach has been particularly impactful in neuroscience, where it has confirmed and helped uncover neural communication elements that define distinct neuronal cell types and elucidate their physiological roles (Fuzik et al., 2016; Huang and Paul, 2019; Paul et al., 2017). Building on this, we leveraged the deep, high-resolution single-cell transcriptomic dataset from Wu et al. to extract key transcriptional features that may help explain and predict the cellular properties of PN subtypes (panel D).

**Limitation:** One consideration in interpreting our analysis is the anatomical distribution and methodological diversity of the datasets used. While some datasets span all axial levels, others are more regionally focused – one from the brachial level and four from the lumbar region – resulting in a relative emphasis on limb-associated DRGs. In addition, the datasets differ in technical aspects, such as the use of single-cell versus single-nucleus approaches, tissue processing protocols, and cell partitioning methods (e.g. droplet-based platforms, FACS, or manual isolation). These factors may contribute to variation in the proportions and types of cells identified. Nonetheless, the datasets show overall concordance. For example, II4-PNs are the most abundant subtype of II-PNs at lumbar levels, with approximately threefold higher representation compared to brachial levels (Wu et al., 2021). Consequently, the other datasets, which are enriched for lumbar DRGs, consistently show a higher proportion of this subtype.

**Box 1—figure 1.**
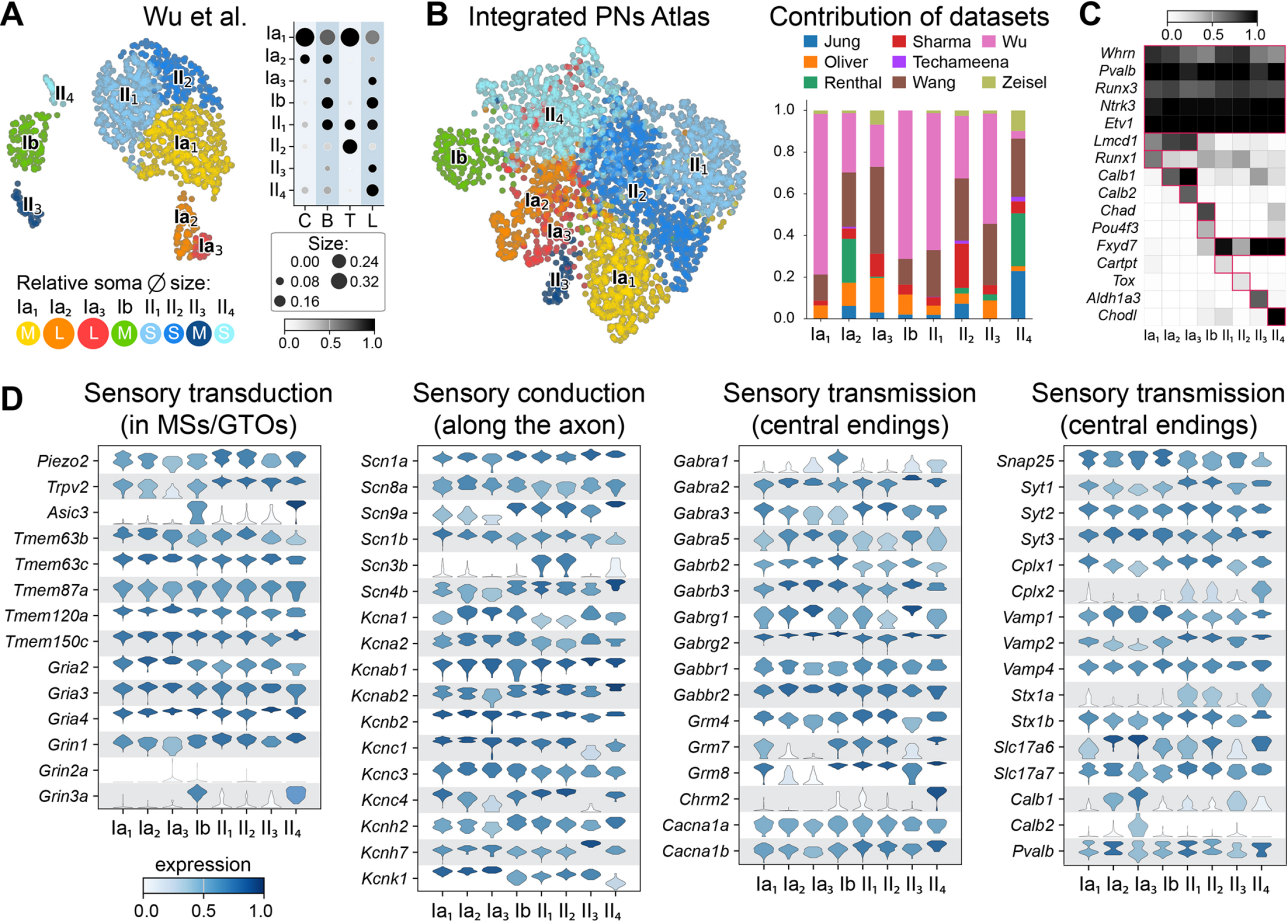
A comprehensive atlas of proprioceptive neuron (PN) cell types and their associated functional features. (**A**) Uniform Manifold Approximation and Projection (UMAP) visualization of single-cell transcriptomes of PNs from Wu et al. dataset, colored by cell type. Below: representation of the soma sizes of the subtypes proportional to the average soma sizes of the respective PN subtypes observed in situ, from Wu et al. Right: dot plot summarizing the rostro-caudal distribution of PN subtypes in dorsal root ganglia (DRG), as in Wu et al. (shown as averages; C: cervical C2; B: brachial C5-8; T: thoracic T1/4/10; L: lumbar L2-5). The size of the circle reflects the percentage of different subtypes among all PNs in each region. The color intensity reflects how a subtype is distributed along the rostro-caudal axis, where 1 represents its highest representation. (**B**) Integration of PN sc/snRNAseq datasets. Left: UMAP of an integrated PNs atlas derived from eight distinct datasets. Right: bar graph depicting the datasets contribution to the integrated atlas (see Materials and methods). (**C**) Heatmap showing marker gene expression for cell types in the integrated PNs atlas. (**D**) Stacked violin plot showing the expression of sensory neuron operational components across PN cell types, based on the Wu et al. dataset.

PNs are classified into eight distinct clusters, all of which express common PN markers, including *Whrn* (whirlin), *Pvalb* (parvalbumin), *Runx3*, *Ntrk3* (TrkC), and *Etv1*, along with cluster-specific genes (Box 1—figure 1C). These clusters can be grouped into three main categories based on highly specific markers identified through an unbiased approach: clusters 1–3 as Ia-PNs (Ia_1-3_, LMCD1^+^), cluster 4 as Ib-PNs (POU4F3^+^/CHAD^+^), and clusters 5–8 as II-PNs (II_1-4_, FXYD7^+^) (Box 1—figure 1C). While on average, the size of the PNs in these three main groups is consistent with previous findings, where primary afferents generally have a larger soma diameter compared to secondary afferents, there is a significant variation in soma size within each group (Box 1—figure 1A). This variability, along with overlap between the groups, complicates classification based solely on cell or axon diameter (Matthews, 1972). Additionally, this suggests that larger axon diameters, higher dynamic sensitivity, and fast conduction velocity are not exclusive features of Ia afferents (Erlanger and Gasser, 1930). For instance, II_3_-PNs have a larger average soma size than Ia_1_-PNs (Wu et al., 2021). This suggests that these traits may also be closely linked to the specific muscles being innervated, and not the afferent type alone. Notably, within each major group, the subtypes displaying the largest soma size, suggestive of heightened dynamic sensitivity, are consistently identified in PNs of the cervicobrachial and lumbar levels that innervate limb muscles (Wu et al., 2021). Hence, the large size Ia_2-3_-, Ib-, and II_3_-PNs are highly restricted to the limb levels, which suggests that they could be specialized in transmitting proprioceptive feedback mostly from limb hypaxial muscles. In support of this, the central endings of calbindin^+^ Ia_2-3_-PN fibers are systematically found innervating the motor neurons of the lateral motor column area, which innervate the limb, and not the medial motor column innervating the epaxial muscles (Wu et al., 2021). Moreover, the Ia_3_-PNs, expressing both calbindin and calretinin, can be distinguished from the Ia_2_-PNs by their particularly larger size and a distinctive innervation pattern. In the forelimb, Ia_3_-PNs preferentially target dorsodistal muscles (including the extensor carpi radialis), primarily serving as extensors of the wrist and digits (Wu et al., 2021). In addition, in bag and chain intrafusal fibers, Ia_3_-PNs specifically coil around the nuclear bag fibers, which are more responsive to the dynamic changes in muscles. Altogether, this strongly suggests further specialization of PN subtypes with potential functional consequences.

Interestingly, the morphological and anatomical features of PN subtypes exhibit a certain correlation with the clustering structure of the transcriptomic data analysis, as the largest subtypes consistently occupy an off-centered position in the Uniform Manifold Approximation and Projection (UMAP) (Box 1—figure 1). Hence, it is possible that certain traits of the subtypes – potentially associated with cell size, dynamic properties, or the target tissue being innervated – could be linked to gene expression differences, as observed during development (Dietrich et al., 2022; Poliak et al., 2016). Together with influencing the spatial arrangement within the clustering observed in the UMAP plot, these differences could carry evident physiological implications. For instance, Ia_3_-PNs exhibit a high expression of genes associated with energy metabolism, indicating higher energy demand for this particularly large size subtype (Wu et al., 2021).

## Functional molecular diversity of PNs

**Figure 2.**
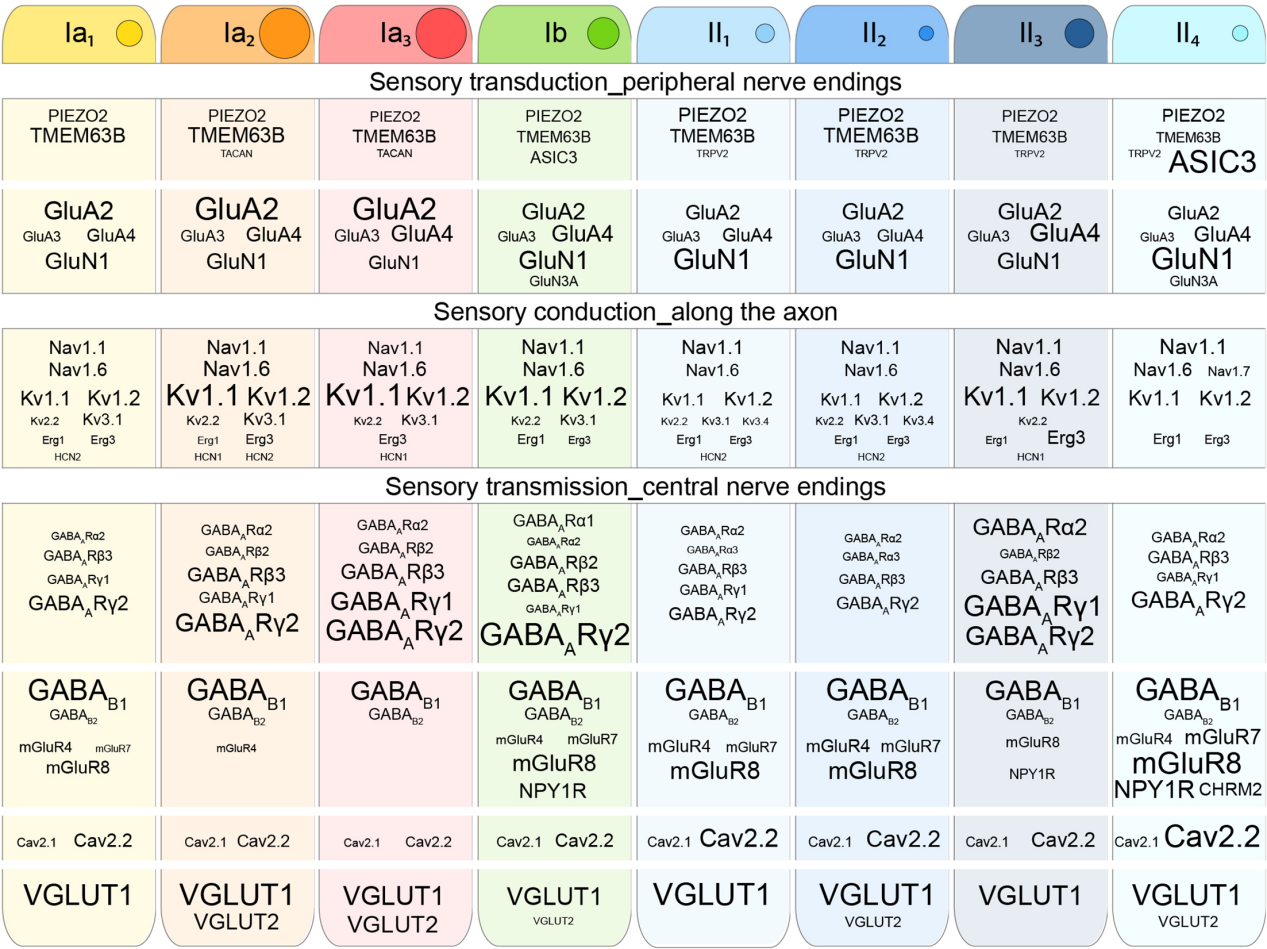
Key molecular markers defining the functional properties of proprioceptive neuron (PN) subtypes. This schematic shows representative genes central to the core functions of proprioceptive neurons (PNs), including sensory transduction, axonal conduction, and synaptic transmission. The selected markers are pivotal for neuronal functionality and were chosen for their high and specific expression. Font size is directly proportional to gene expression levels, normalized within each functional category to the most highly expressed gene. Functional categories are separated by white lines. The size of the circle next to each PN subtype name reflects its relative somatic diameter as observed in vivo (Wu et al., 2021).

Although single-cell analysis data cannot provide a direct readout of cell phenotypes, prior studies have shown its utility in forming an emerging picture of cell identities, allowing us to predict or infer their biological properties (Fuzik et al., 2016; Huang and Paul, 2019; Ofengeim et al., 2017; Paul et al., 2017; Petitpré et al., 2020; Petitpré et al., 2018). This conceptual framework, when considered alongside previous histological and functional studies, can enhance our understanding of neuron-type physiology and function. In this section, we delve into the transcriptional architecture underlying the neuronal communication properties of PN subtypes, integrating this knowledge with recent functional data on PNs physiology and function. While not aiming to provide a definitive or comprehensive account, it offers a tentative overview, grounded in prior research and high-resolution molecular analyses (see Figure 2 and box panels for molecular references used in this section).

**Figure 2—figure supplement 1.**
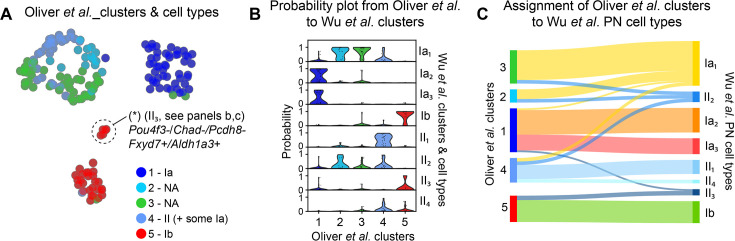
Corresponding cell types between datasets. (**A**) Uniform Manifold Approximation and Projection (UMAP) plot of proprioceptive neurons (PNs) from Oliver et al. with Wu et al. nomenclature. NA: non-assigned. (*) This cluster is identified as II_3_-PNs after subclustering and cluster assignment; cells of this cluster do not express marker genes of the Ib population. (**B**) Stacked violin plot of cell-type probability scores of cells from Oliver et al. predicted as Wu et al. cell types. (**C**) Sankey plot representing the cluster and cell correspondence from Oliver et al. to Wu et al. nomenclatures.

### Sensory transduction in PNs

In PNs, the initiation of sensory transduction is primarily triggered by mechanically induced currents resulting from the stretch of their nerve endings.

#### PIEZO2, ASIC, and TRP channels

The principal mechanosensitive ion channel in PNs is PIEZO2 (Chesler et al., 2016; Woo et al., 2015). It shows a high, similar expression across all PN subtypes and is responsible for the majority of mechanically activated (MA) currents in PNs. However, studies have suggested the existence of additional mechanosensors in PNs, as PIEZO2 channels specifically account for rapidly adapting currents and do not impact intermediately or slowly adapting currents (Hunt and Ottoson, 1975; Matthews, 1972), which also characterize PN physiology. These include members of the TRP (transient receptor potential) channel protein family, which display differential expression among PN subtypes, with high expression of *Trpv2* in secondary afferents (Tamura et al., 2005; Wu et al., 2021). While classified as mechanosensitive cation channels (Ranade et al., 2015), the direct role of TRP proteins as force-gated channels remains unclear. They have been proposed to regulate the sensitivity of the mechanotransducer complex; their expression in PNs could therefore have a potential impact on the quality of proprioceptive feedback, as demonstrated in *Drosophila* (Cheng et al., 2010). An additional mechanosensitive ion channel, or constituent of mechanotransduction channels, is ASIC3 (Lin et al., 2016). It plays a role in finely adjusting the activation of PN afferents, with predominant expression observed limb level-enriched II_4_-PNs, and in Ib-PNs. Its relevance for proprioceptive feedback was clearly observed in dark conditions, in which conditional deletion of Asic3 in PV-positive cells led to motor deficits in grid walking and balance-beam behavioral tasks (Lin et al., 2016). Various studies have, however, questioned its direct role as mechanical transducers in DRG neurons (Drew et al., 2004). Instead, ASICs may mediate fast excitatory synaptic transmission at junctions between PN afferent endings and intrafusal fibers, involving a transmitter release mechanism similar to that suggested for Merkel cell-neurite complexes (Yamada et al., 2024). This would imply that intrafusal fibers actively participate in muscle stretch responses beyond passive mechanical coupling, akin to the functional connection between Merkel cells and Aβ-afferent nerves and the activation of Merkel cells via PIEZO2, which plays a role in generating slowly adapting responses (Maksimovic et al., 2014; Woo et al., 2014). Interestingly, intrafusal bag fibers also express PIEZO2 (Kim et al., 2020) and might be stretch-sensitive, suggesting a dual-receptor system (intrafusal fibers and sensory afferents) for controlling the temporal acuity of proprioception from MSs. Finally, a recent study revealed that acidosis during fatiguing exercise activates ASIC3-expressing PN endings in muscle, proposing a mechanism in which glutamate release primes nociceptive afferent endings (Lee et al., 2025). Notably, this priming is disrupted by substance P, whose receptor, Tacr1, is also selectively expressed by II_4_-PNs (Wu et al., 2021*).* These findings suggest that specific proprioceptor subpopulations may contribute to muscle soreness sensation.

#### TMEM membrane proteins

Members of the TMEM gene family (Beaulieu-Laroche et al., 2020; Murthy et al., 2018), among which *Tmem63b*, exhibit high expression in all PNs with varied expression between subtypes. Other members, such as *Tmem120a*, *-63c*, *-150c*, or *-87a*, are also expressed but at lower levels. Among these, TMEM150C (Tentonin 3) was identified in MS afferents in direct contact with intrafusal muscle fibers and has been demonstrated to be essential for the slowly adapting current of PNs and for motor coordination (Bewick and Banks, 2021). Yet, the exact mechanism through which it regulates PN activation remains unclear. TMEM150C has been shown to induce MA current with slow inactivation kinetics when overexpressed heterologously (Hong et al., 2016), and recent in vitro findings suggest its potential interaction with mechanosensitive ion channels from different classes, such as PIEZO2 (Anderson et al., 2018). This interaction could extend the duration of the MA current and decrease the activation threshold of these channels (Wilkinson, 2022). A recent study identified ELKIN1 (*Tmem87a*) as a mechanically gated ion channel required for normal touch sensitivity in mice (Chakrabarti et al., 2024). Although expressed at low levels in all PNs, its functional role in proprioception remains unclear. Given that *Tmem87a*-deficient mice retain normal locomotion and some withdrawal reflexes (Chakrabarti et al., 2024), any potential contribution is likely to be limited to specific motor contexts.

#### Conformation and sensitivity of nerve endings

Another factor to consider in modulating the sensitivity of PNs afferent endings to mechanical stimuli is the biophysical aspect of their three-dimensional arrangement within the peripheral sensory organs. The structural diversity of each type of nerve ending is crucial for finely tuning the sensitivity of afferents within their peripheral end organ, leveraging the unique activation characteristics of mechanosensitive ion channels. For instance, PIEZOs are large, homotrimeric membrane proteins arranged as a triskelion with three curved arms deforming a large membrane area around them to create an inward-curving dome (Haselwandter et al., 2022; Mulhall et al., 2023). This structure makes the PIEZO channels highly sensitive to lateral membrane tension. The mechanosensitivity of nerve terminals largely depends on the bending elastic properties of the cell membrane, determining how external forces – the mechanical stimuli – translate into its deformation. Hence, the difference in the ultrastructural organization between primary and secondary endings on the MS intrafusal fibers could have a functional impact. The spiral terminations of primary endings on intrafusal fibers, with fewer sprays, align precisely with the stretch axis of the fibers and are presumed to fit a maximum length of nerve endings and, consequently, a potentially higher number of mechanosensitive channels. Considering the region of MS innervation, where primary afferents innervate the equatorial region of both bag and chain intrafusal fibers, while secondary afferents are predominantly located on the poles of chain fibers only, and factoring in the distinct intrinsic properties of intrafusal fibers along their length and between bag and chain fibers, these variations might play a crucial role in the sensitivity and adaptability properties of various proprioceptive afferents innervating MSs (Matthews, 1972). This includes Ia_3_-PNs, which exclusively innervate bag fibers at the forelimb level (Wu et al., 2021).

#### Macrophages, a new cellular player in proprioception

Resident MS macrophages, located near PN afferents, have been found to exert a direct influence on their activity (Yan et al., 2025). Their activation prompts the stimulation of sensory afferents and the stretch reflex arc through rapid glutamatergic signaling, occurring within milliseconds. The significance of these macrophages could be particularly evident during periods of heightened metabolic demand, by facilitating the sustained and optimized activity of proprioceptive feedback from muscles engaged in prolonged activity. It remains to be determined whether a similar system operates within the GTO. This discovery not only enhances our understanding of the precise regulation of muscle sensorimotor feedback but also sheds light on the significance of interactions between neuro-immune systems in the physiology of sensory circuits.

### Sensory conduction

#### Sodium channels

Voltage-gated sodium channels (VGSCs) are vital for initiating and propagating action potentials in excitable cells (Vacher et al., 2008). In mammals, VGSCs are complex assemblies comprising α and β subunits. The α subunit forms the ion-conducting pore and associates with two different β subunits (Vacher et al., 2008). Among the VGSC α subunits, Nav1.1 (*Scn1a*) and Nav1.6 (*Scn8a*), prominently expressed in large mechanosensitive neurons within the DRG, are abundantly and ubiquitously expressed across PN subtypes (Espino et al., 2022; Fukuoka et al., 2008; Wu et al., 2021; Zeisel et al., 2018). Compared to Nav1.8 and Nav1.9, which are specifically expressed in DRG C-fiber neurons, including small-diameter nociceptors and, to a lesser extent, C-LTMRs (Fukuoka et al., 2008; Zeisel et al., 2018; Zheng et al., 2019), Nav1.1 and Nav1.6 open more rapidly and at more negative membrane potential and exhibit faster kinetics.

Nav1.1 is primarily found in nerve terminals rather than the nodes of Ranvier in PNs (Espino et al., 2025). Its absence in sensory neurons leads to irregular static firing at the afferent level while maintaining dynamic firing, resulting in uncoordinated movements and abnormal limb positioning (Carrasco et al., 2017; Duflocq et al., 2008; Espino et al., 2022). Therefore, Nav1.1 does not appear to have a generalized role in maintaining high-frequency firing, but a more specific contribution to muscle-afferent static sensitivity.

In contrast, Nav1.6 is localized at the nodes of Ranvier in PNs (Carrasco et al., 2017; Chen et al., 2018). It exhibits unique biophysical properties, including activation at more negative membrane potentials, increased levels of persistent and resurgent currents, and a higher frequency of repetitive neuronal firing (Raman et al., 1997; Rush et al., 2005). These characteristics make Nav1.6 a favorable key regulator of dynamic firing in PNs, as previously suggested in vitro (Chen et al., 2018). Its absence in DRG neurons in vivo, however, prevented proprioceptive neurotransmission for both dynamic and static muscle movement (Espino et al., 2025), suggesting a more general role than dynamic sensitivity per se. However, given the important non-cell-autonomous role of Nav1.6 in the proper development of MSs and skeletal muscle fibers (Espino et al., 2025), future studies will be necessary to disentangle and specifically address the cell-autonomous contribution of this ion channel to PN properties in the adult. Nav1.7 (Scn9a), another rapidly inactivating channel, is widely expressed in most DRG neurons and is essential for pain sensation (Cox et al., 2006), but typically absent from PNs (Zeisel et al., 2018). However, it is present in a subtype-specific manner, being particularly prevalent in II_4_-PNs, supporting previous Nav1.7 immunolabeling (Black et al., 2012). Yet, despite the potential complementary roles of Nav1.1 and Nav1.6 in regulating static and dynamic firing patterns in PNs (Espino et al., 2022; Espino et al., 2025), which is certainly in line with their neuronal localization, the potential involvement of Nav1.7 in non-Ia-PNs, especially in the small II_4_-PNs, remains uncertain.

In contrast to the α subunits of VGSCs, the β subunits play a role in regulating both channel trafficking, including clustering to specific neuronal domains, and the biophysical properties of the channels (Chahine et al., 2005; Isom, 2001). Although findings from null mouse models indicate subtle and cell type-specific effects of β subunits on sodium currents in vivo, they exert significant effects on overall cellular excitability (Aman et al., 2009; Brackenbury et al., 2010; Chen et al., 2004).

Navβ1 (*Scn1b*) is preferentially expressed at high levels in large diameter neurons, including all PNs, within the DRG. Its localization at nodes of Ranvier in peripheral myelinated nerves suggests a role in axonal conduction. Co-expression of Navβ1 subunits with α subunits enhances the peak sodium current, accelerates its inactivation, and shifts the voltage dependence of inactivation to more negative membrane potentials, increasing the fraction of channels operating in the fast-gating mode. Navβ3 (*Scn3b*) has a milder effect on the fast-gating mode of α subunits (Morgan et al., 2000) and is expressed only in II_1,2_-PNs, potentially modulating their firing frequency differently.

Navβ4 (*Scn4b*) is also expressed in PNs, with particularly high levels in non-Ia-PNs, resembling the subtype-specific expression pattern of Nav1.7. These observations suggest that most PNs operate in a fast-gating mode due to interactions between α subunits and β1 or β4 subunits, enabling high-frequency firing (Lv et al., 2024; Ulbricht, 2005). II_1,2_-PNs might adopt a more moderate gating mode depending on the presence of β3 subunits in their VGSC configuration.

#### Potassium channels

The firing patterns of neurons are influenced by the expression of various members of the voltage-gated potassium channel (VGKC) family, where α subunits assemble into tetramers to form the conductance pore. These channels, along with their auxiliary subunits that modulate Kv channel activity, display diverse expression patterns across different subtypes of peripheral neurons, likely contributing to the regulation of their firing properties. Previous studies have highlighted the significance of Kv1 channels in DRG myelinated nerve fibers, particularly Kv1.1 and Kv1.2 (Zeisel et al., 2018; Zheng et al., 2019), which are prominently localized at the axon initial segments and juxtaparanodal regions adjacent to the nodes of Ranvier along their axons (Chien et al., 2007; Rasband, 2010; Rasband and Peles, 2016; Rasband et al., 1998). This localization enhances action potential fidelity at the nodes (Lai and Jan, 2006) and suggests the formation of Kv1.1/Kv1.2 heteromers with intermediate biophysical properties (Dodson et al., 2002; Rasband et al., 1998). Notably, Kv1.1 and Kv1.2 are predominantly expressed in large diameter, limb-innervating PNs (including Ia_2,3_-, Ib-, and II_3_-PNs), and their activity in vitro has been linked to the phasic properties of a specific group of PNs (Oliver et al., 2021), suggesting a potential role in supporting the dynamic sensitivity of these afferents.

The auxiliary subunits Kvβ1 and Kvβ2 are broadly expressed in PNs, with Kvβ2 showing higher expression, particularly in II_4_-PNs, and likely facilitating the trafficking and surface expression of heteromeric complexes (Manganas and Trimmer, 2000). These findings suggest that within the juxtaparanodal regions of the nodes of Ranvier, Kv1.1/Kv1.2 channels may play a role in quickly repolarizing the axon, thereby facilitating the rapid conduction of high-frequency action potentials. Additionally, it is anticipated that a combination of Kv1.1/Kv1.2 channels with the Kvβ1 subunit in the presynaptic region could potentially promote synaptic facilitation and contribute to enhancing information transfer during high-frequency stimulation (Cho et al., 2020; Wang et al., 1993).

The generation of high-frequency action potentials by PNs suggests their expression of VGKCs from the Kv3 family (Zheng et al., 2019), traditionally linked to the capacity of certain neurons to fire action potentials rapidly and release neurotransmitters at high rates, primarily localizing in axon terminals (Kaczmarek and Zhang, 2017). Previous bulk-sequencing studies have revealed the expression of *Kcnc1* (Kv3.1) in PNs, along with low levels of *Kcnc3* (Kv3.3) and *Kcnc4* (Kv3.4) (Zheng et al., 2019), though their expression profiles vary significantly depending on the PN subtype.

Kv3.1 is preferentially expressed in all Ia- and Ib-PNs, with lower levels in II_1,2_-PNs and minimal presence in II_3,4_-PNs. Conversely, Kv3.4 is highly expressed in PNs innervating axial muscles such as Ia_1_- and II_2_-PNs, while being less abundant or absent in those innervating limb muscles such as Ia_2,3_- and II_3_-PNs, consistent with previous findings indicating expression in approximately 50% of parvalbumin-positive DRG neurons (Chien et al., 2007). As with all Kv channels, functional Kv3 channels are tetramers of four Kv subunits. Therefore, Ia_2,3_-PNs and Ib-PNs primarily express Kv3.1-only tetramers, potentially contributing to the rapid repolarization characteristic of these dynamic populations, facilitating high-frequency action potential firing. Histologically, Kv3.1 has been identified in axon terminals and at nodes of Ranvier (Trimmer, 2015). However, it remains unclear why II_3_-PNs, despite being large and exclusively found at limb levels, exhibit minimal expression of Kv3 channels, relying predominantly on Kv1.1/Kv1.2 channels to regulate their firing properties.

Kv2.2 is expressed across all PN subtypes, albeit at modest levels, and is found in axons at paranodes, adjacent to Kv1.1/Kv1.2 channels located at juxtaparanodes (Stewart et al., 2024). Its expression facilitates repetitive firing across various neuronal types (Guan et al., 2013; Liu and Bean, 2014; Tong et al., 2013).

Another potassium channel, independent of voltage and highly expressed in myelinated DRG neurons (Zeisel et al., 2018), particularly in Ia proprioceptive neurons (Ia-PNs), is the two-pore domain background potassium K2P1.1 channel (*Kcnk1*), also known as TWIK-1. TWIK-1, which can be influenced by temperature (Enyedi and Czirják, 2010; Kang et al., 2005; Oakes et al., 2016), contributes to the background potassium conductance in cells, thereby regulating the resting membrane potential and cellular excitability. While its role and localization in the nervous system are not extensively studied, it could potentially enhance the rapid conduction properties of Ia-PNs by aiding rapid action potential repolarization at the nodes along their axons, as observed in other K2P channels in skin LTMR fibers of the trigeminal ganglia innervating the whiskers (Kanda et al., 2019).

Erg channels, unlike other VGKCs, are inward-rectifying potassium channels. Both Erg1 (*Kcnh2*) and Erg3 (*Kcnh7*) are expressed in PNs, exhibiting varying levels of expression across different subtypes and are likely to form heteromers. Their functions are diverse, affecting action potential threshold and frequency accommodation, but also facilitating high-frequency firing (Bauer and Schwarz, 2018; Cui and Strowbridge, 2018). This functional diversity necessitates further investigation in the proprioceptive field to elucidate their role in the firing characteristics of each PN subtype.

#### GABAergic modulation

Presynaptic regulation of proprioceptive inputs onto secondary neurons in the CNS provides an additional layer of control, modulating the gain and filtering of relevant sensory information (Clarac et al., 1992; Eccles et al., 1961; Fink et al., 2014; Frank and Fuortes, 1957; Hari et al., 2022; Tomatsu et al., 2023; Zimmerman et al., 2019). This process involves GABAergic interneurons, which release GABA to activate GABA ionotropic receptors (GABA_A_Rs) on sensory afferents or GABA metabotropic receptors (GABA_B_Rs) on sensory terminals. These interneurons’ activity may be regulated by descending signals or sensory-evoked inputs, forming axo-axonic or axo-sensory endings connections (Hari et al., 2022; Watson and Bazzaz, 2001).

GABA_A_Rs are expressed near the nodes of Ranvier at the branch points of PNs axons within the spinal cord (Hari et al., 2022). Due to the high intracellular chloride concentrations (Wilke et al., 2020), the activation of GABA_A_Rs causes depolarization of sensory axons, preventing conduction failure at branch points and facilitating nodal and reflex responses, presumably across many spinal segments (Hari et al., 2022; Mahrous et al., 2023; Metz et al., 2023). In contrast, activation of GABA_B_Rs at sensory terminals causes presynaptic inhibition, fine-tuning reflex gains locally (Bennett et al., 1996; Eccles and Willis, 1963; Fink et al., 2014).

In mammals, a minimal GABA_A_R requires an α and a β subunit, with the most common ratio being 2α/2β/1γ when a γ subunit is added (Olsen and Sieghart, 2009). Due to the diversity of subunits, numerous GABA_A_R subtypes exist, characterized by their subunit combinations, allowing a single cell to express multiple subtypes, which contributes to functional diversity. Transcriptomic and in situ protein profiling studies show that the most highly expressed GABA_A_R subunits in PNs are *Gabra1/2/3/5* (α1/2/3/5), *Gabrb2/3* (β2/3), and *Gabrg1/2* (γ1/2), with distinct expression among PN subtypes (Hari et al., 2022; Lucas-Osma et al., 2018; Oliver et al., 2021; Wu et al., 2021).

The α1 subunit is uniquely and highly expressed in Ib-PNs, providing fast GABA_A_R kinetics for quicker synaptic responses compared to the α2 (highly expressed in II_3_-PNs) and α3 subunits (expressed in all PNs but Ib) (Gingrich et al., 1995; Picton and Fisher, 2007; Sallard et al., 2021). The α5 subunit, typically found in extrasynaptic GABA_A_Rs, is highly sensitive to GABA and desensitizes slower. It is exclusively expressed in limb-innervating PNs, including Ia_2-3_, Ib, and II_3-4_, suggesting a role in priming the limb sensorimotor system for external perturbations through slow tonic depolarization (Hari et al., 2022; Lin et al., 2023).

The β2 subunit, enriched in Ib-PNs, associates with the α1 subunit to contribute to the fast kinetics of GABA_A_Rs (Sallard et al., 2021). The β3 subunit, enriched in large limb-innervating PNs (Ia_2-3_, Ib, II_3-4_), associates with α2/3 subunits, reducing postsynaptic current decay kinetics (Nguyen and Nicoll, 2018; Ramadan et al., 2003; Sallard et al., 2021). The γ2 subunit, which is preferentially synaptic and abundantly expressed in the nervous system, is highly expressed in PNs, particularly large limb-innervating PNs. The γ1 subunit, mainly extrasynaptic, is found predominantly in large limb-specific Ia_2-3_- and II_3_-PNs. Together, this suggests a higher proportion of synaptic GABA_A_Rs overall and an enrichment of extrasynaptic GABA_A_Rs in Ia_2-3_- and II_3_-PNs. This would imply that MS-specific limb PNs with larger soma diameters and high conduction velocities can be targeted by tonic activation, tuning them to a lower threshold. As a result, this would enhance their sensitivity to muscle stretch and facilitate long-lasting MS afferent responses through cortical or sensory conditioning (Hari et al., 2022; Metz et al., 2023).

In contrast, activation of GABA_B_Rs in proprioceptive axon terminals primarily causes presynaptic inhibition (Eccles and Willis, 1963; Hari et al., 2022), reducing action potential frequency and neurotransmitter release (Chalifoux and Carter, 2011; Sakaba and Neher, 2003; Takahashi et al., 1998). GABA_B_Rs are heterodimers of GABA_B1_ and GABA_B2_ subunits, coded by *Gabbr1* and *Gabbr2*, respectively (Gassmann and Bettler, 2012). Unlike GABA_A_R subunits, GABA_B_R subunits appear to be similarly expressed across all PN subtypes, indicating a widespread potential for presynaptic inhibition among PNs.

#### Glutamatergic modulation

Metabotropic glutamate receptors (mGluRs) are also critical modulators of neuronal activity and plasticity, potentially influencing these processes at postsynaptic sites, peripheral nerve endings, or presynaptically within the spinal cord. Transcriptomic analyses indicate that group III mGluRs, which couple with Gα_i/o_ to mediate neuronal inhibition – specifically *Grm4*, *Grm7*, and *Grm8* (mGluR4, mGluR7, and mGluR8) – are predominantly expressed in PNs. Evidence suggests that group III mGluRs play an activity-dependent autoinhibitory role in peripheral endings of nociceptor afferents, facilitating desensitization during prolonged stimulation (Carlton et al., 2011). Additionally, these receptors are present presynaptically at primary gustatory afferent synapses, where their activation inhibits glutamate release (Hallock et al., 2009). Furthermore, mGluR7 has been localized to central terminals of nociceptive afferents (Li et al., 1997). These findings underscore the need for further research to elucidate mGluR expression in PNs and their physiological roles in proprioceptive afferents during movement.

Additional metabotropic receptors are expressed in PNs and include the muscarinic acetylcholine receptor M4 (*Chrm4*) and the neuropeptide Y receptor type 1 (*Npy1r*) (Oliver et al., 2021; Wu et al., 2021). CHRM4 expression appears restricted to II_4_-PNs, whereas NPY1R is primarily found in II_4_- and Ib-PNs and more sparsely in II_3_-PNs. Both receptors are coupled to Gαi/o proteins (Langmead et al., 2008; Park et al., 2022), and their activation typically leads to neuronal inhibition, suggesting a potential role in modulating sensory gain in specific proprioceptive subtypes.

Neuropeptide signaling is traditionally associated with nociceptive pathway in the somatosensory neurons. The possibility that neuropeptides – generally considered slow, modulatory signals – may directly modulate proprioceptive afferents introduces a novel conceptual layer. Strikingly, some PNs themselves express neuropeptides; e.g., substance P (*Tac1*) is present in all type II afferents except the large II_3_-PNs (Oliver et al., 2021; Wu et al., 2021). This raises the possibility that proprioceptors may actively shape local or central circuit function.

### Sensory transmission

The molecular composition of the presynaptic machinery, that comprises voltage-gated calcium channels (VGCCs) at the nerve terminal (Dittman and Ryan, 2019; Dolphin and Lee, 2020; Eggermann et al., 2011; Südhof, 2012) and proteins of the SNARE (soluble N-ethylmaleimide-sensitive factor attachment protein [SNAP] receptors) complex (Südhof and Rothman, 2009), is crucial for the timing and regulation of synaptic outputs.

#### Calcium channels

VGCCs, also known as Cav channels, are composed of a pore-forming α1 subunit and auxiliary β and α2δ subunits. This composition varies between synapses, leading to diversity in calcium dynamics and synaptic release (Zhang et al., 2022a). Fast neurotransmission primarily relies on calcium influx through Cav2 channels. In PNs, the P/Q-type Cav2.1 channel is uniformly expressed, while the N-type Cav2.2 channel shows varied expression levels inversely correlated with cell size, with the lowest levels in Ia_2-3_ neurons. Typically, Cav2.1 channels cluster closer to vesicles, forming nanodomains of elevated intracellular calcium at the active zone, supporting rapid and precise glutamate release, especially during high-frequency stimulation (Dolphin and Lee, 2020; Ishikawa et al., 2005; Kusch et al., 2018). Cav2.2 channels, usually surrounding the active zone, may contribute less to fast neurotransmitter release and be more active during action potential broadening and lower firing rates (Li et al., 2007).

#### The pre-synaptic fusion machinery

Differential expression of molecules in the SNARE complex (including syntaxins, synaptobrevins, and SNAP-25) and of synaptotagmins contributes to variations in synaptic coding. Among the seventeen isoforms of mammalian synaptotagmins, eight (1–3, 5–7, 9, and 10) function as calcium sensors. Most of these sensors are located at presynaptic terminals, where they are crucial for Ca^2+^-dependent neurotransmitter release by coupling Ca^2+^ influx to SNARE proteins, facilitating the exocytosis (Südhof, 2002; Wolfes and Dean, 2020).

Among the synaptotagmin calcium sensors, Syt 2 is particularly noteworthy. It is localized to synaptic vesicles and exhibits the fastest onset and decline in release (Südhof, 2002; Sugita et al., 2002; Xu et al., 2007). Syt 2 is widely expressed in all PNs, presumably playing a crucial role in rapid and synchronous synaptic vesicle exocytosis. Due to their lower Ca^2+^ affinity, synapses expressing Syt 2 require higher Ca^2+^ concentrations for vesicle release, achievable locally through Cav2.1 channels (see above). Large PNs also express endogenous Ca^2+^ buffers such as parvalbumin (in all PNs), calbindin, and calretinin (in Ia_2-3_), enhancing synaptic efficiency by reducing asynchronous release during high activity rates (Eggermann et al., 2011; Zhang et al., 2022b). This specific expression in Ia_2-3_-PNs may help regulate the amplitude and dynamics of Ca^2+^ nanodomains and their coupling with rapid vesicle release machinery (Eggermann et al., 2011). Another fast synaptotagmin Ca^2+^ sensor, Syt 1, is found in PNs, with higher expression in smaller neurons. Its intermediate kinetic properties could slightly extend the timing of the vesicle release event (Sugita et al., 2002).

Syt 3, another Ca^2+^ sensor, is highly expressed in all PNs. Unlike Syt 1 and Syt 2, Syt 3 is localized to the plasma membrane and has a 10-fold higher affinity for Ca^2+^. Previous studies indicate that Syt 3 mediates the slow component of synaptic release (Sugita et al., 2002). Recent research has highlighted the importance of Syt 3 also in the postsynaptic membrane for driving Ca^2+^-dependent endocytosis of AMPA receptors, promoting long-term depression of synaptic strength (Awasthi et al., 2019). Considering the glutamatergic signaling between resident macrophages and peripheral endings of PNs in MSs and the expression of AMPA receptors (*Gria2-4*) by PNs, Syt 3 could fine-tune the modulatory action of macrophages on PN afferents and/or prevent AMPA-mediated overstimulation and excitotoxicity at their peripheral nerve endings (Wang et al., 2012; Yan et al., 2025; Zhai et al., 2023).

#### Glutamate transporters

Finally, while most studies traditionally depict PNs as exclusively expressing vesicular glutamate transporter 1 (VGLUT1), recent research has shown that the VGLUT2 isoform is also highly expressed in Ia_2-3_ PNs, and to a lesser extent in II_2_ and II_4_ PNs (Landry et al., 2004; Scherrer et al., 2010; Wu et al., 2021). This dual expression suggests a higher release probability at their synapses and indicates distinct characteristics of short-term plasticity (Nakakubo et al., 2020; Weston et al., 2011).

## Conclusions

Since the discovery of proprioceptive afferents over a century ago, there has been growing appreciation that the proprioceptive system probably operates through subpopulations of neurons beyond their classic categorization into Ia, Ib, and II-PNs (Matthews, 1972; Sherrington, 1910). In recent years, advances in single-cell technologies have provided evidence for this phenotypic diversity, which is covered in this review, further supporting the notion that the proprioceptive system comprises diverse sensory channels essential for transmitting the various features of muscle activities throughout the body. This diversity indicates subdivisions between functional subtypes rather than variations within continuous populations of neurons, posing conceptual challenges when defining PN subtypes at a fine resolution. For instance, PNs exhibit increased activity and lasting epigenetic and gene expression changes when exposed to sustained environmental changes, such as long-term voluntary exercise (Hutson et al., 2019). This robust neuroplasticity has been proposed to involve certain Ia_1_-PNs transitioning to a Ia_2_-like phenotype, characterized by increased size, which is suggested to underlie enhanced dynamic properties (Wu et al., 2021). A crucial question, therefore, is whether and how this phenotype switching would contribute to the sensorimotor function improvements typically observed after motor training (Aman et al., 2014; Pahor et al., 2014; Petzinger et al., 2013; Yilmaz et al., 2024) and if these changes persist long term (Hutson et al., 2019). This phenomenon also raises important questions about the stability of sensory neuron identity, suggesting that these neurons can exhibit plasticity in response to sustained environmental changes that are not limited to neuropathological conditions, as seen with chronic pain and tinnitus in the pain and auditory pathways (Finnerup et al., 2021; Shore and Wu, 2019; Techameena et al., 2024).

Finally, with advances in understanding the molecular identities and diversity of PNs, a crucial next challenge is to decipher how these neurons integrate centrally within the spinal cord and brainstem circuits to control fine motor behavior. This research will have the potential to unveil intricate neural mechanisms that govern motor control, bridging the gap between motor functions and sensory processing.

## Materials and methods

### Data acquisition and preprocessing

The count matrices and associated metadata were downloaded from the accession listed in the table below. The analysis of single-cell/nuclei RNA-seq was done using Scanpy (Wolf et al., 2018) (v1.10.2) package in Python3 with default parameters unless otherwise specified. The preprocessing of the data was done according to the methods described in the original papers.

**Table:** 

Source	Method	Accession	Region
Wu et al., 2021	Smart-seq2	GSE156180	C5-T1
Oliver et al., 2021	Smart-seq	GSE162263	All axial levels
Renthal et al., 2020	inDrops	GSE154659	L3-5
Wang et al., 2021	10×	GSE155622	L4-5
Sharma et al., 2020	10×	GSE139088	All axial levels
Jung et al., 2023	10×	GSE201654	L1-6
Techameena et al., 2024	10× & Smart-seq3xpress	GSE253345	L4-6
Zeisel et al., 2018	10×	SRP135960	All axial levels

### Identification of proprioceptors

All cells were obtained from adult, non-injured (control) animals and were identified as PNs for each dataset using the combined expression of *Pvalb, Runx3*, *Ntrk3*, *Etv1*, and *Whrn* genes if the dataset was not already provided with cell-type annotation metadata. To elaborate on this process, the cells were clustered using the Leiden algorithm with resolution equal to 3. Then, the score of the genes previously mentioned was calculated for the cell with the ‘score_genes’ function in Scanpy. Clusters with a score above zero and in the top 10 percentile were identified as proprioceptors.

### Initial integration

The initial integration was done on Wu et al. and Oliver et al. datasets aiming to unify the nomenclatures between the two datasets. To accomplish this, the integration was done on the cells using scVI integration workflow (Gayoso et al., 2022) on the top 3000 highly variable genes computed by ‘highly_variable_genes’ with parameter batch_key = ‘Source’, flavor = ‘seurat_v3’, and layer = ‘counts’. After identifying highly variable genes, we trained the scVI model for unsupervised integration to correct for batch effects and other covariates such as sequencing technologies, percent mitochondrial genes, and percent ribosomal genes. We modeled that the distribution of gene expression followed a negative binomial, and the integration model was trained for 400 epochs with two layers. Once scVI was trained, we initialized the scANVI model (Xu et al., 2021) using the weights from the pretrained scVI model to perform semi-supervised integration and label transfer of Wu et al. nomenclature to cells from Oliver et al. scANVI model was trained until converged.

### Integration of all PNs

The integration in this part followed the same logic and parameters as the initial integration process, but here the integration was extended to proprioceptors from every data source listed in the table below. Briefly, the scVI model was trained for 400 epochs with three layers on the cells, and then the scANVI model was initialized using the pretrained weights from the scVI model. Finally, we assessed the quality of dataset integration by examining the expression patterns of canonical markers for each PN subtype.

## Data Availability

The interactive visualization of the atlases will be accessed through the CELLxGENE platform from Chan Zuckerberg Initiative, as "Muscle Proprioceptive Neurons Atlas".
